# Cannabis- and HIV-related perturbations to the cortical gamma dynamics supporting inhibitory processing

**DOI:** 10.1093/braincomms/fcaf190

**Published:** 2025-05-15

**Authors:** Mikki Schantell, Mia C Lulli, Kellen M McDonald, Lucy K Horne, Jason A John, Anna T Coutant, Hannah J Okelberry, Ryan Glesinger, Yasra Arif, Jennifer L O’Neill, Sara H Bares, Pamela E May-Weeks, Tony W Wilson

**Affiliations:** Institute for Human Neuroscience, Boys Town National Research Hospital, 14090 Mother Teresa Ln., Boys Town, NE 68010, USA; College of Medicine, University of Nebraska Medical Center (UNMC), Omaha, NE 68198, USA; Institute for Human Neuroscience, Boys Town National Research Hospital, 14090 Mother Teresa Ln., Boys Town, NE 68010, USA; Institute for Human Neuroscience, Boys Town National Research Hospital, 14090 Mother Teresa Ln., Boys Town, NE 68010, USA; Department of Pharmacology and Neuroscience, Creighton University, Omaha, NE 68178, USA; Institute for Human Neuroscience, Boys Town National Research Hospital, 14090 Mother Teresa Ln., Boys Town, NE 68010, USA; Institute for Human Neuroscience, Boys Town National Research Hospital, 14090 Mother Teresa Ln., Boys Town, NE 68010, USA; Institute for Human Neuroscience, Boys Town National Research Hospital, 14090 Mother Teresa Ln., Boys Town, NE 68010, USA; Institute for Human Neuroscience, Boys Town National Research Hospital, 14090 Mother Teresa Ln., Boys Town, NE 68010, USA; Institute for Human Neuroscience, Boys Town National Research Hospital, 14090 Mother Teresa Ln., Boys Town, NE 68010, USA; Institute for Human Neuroscience, Boys Town National Research Hospital, 14090 Mother Teresa Ln., Boys Town, NE 68010, USA; Division of Infectious Diseases, Department of Internal Medicine, University of Nebraska Medical Center (UNMC), Omaha, NE 68198, USA; Division of Infectious Diseases, Department of Internal Medicine, University of Nebraska Medical Center (UNMC), Omaha, NE 68198, USA; Department of Neurological Sciences, University of Nebraska Medical Center (UNMC), Omaha, NE 68198, USA; Institute for Human Neuroscience, Boys Town National Research Hospital, 14090 Mother Teresa Ln., Boys Town, NE 68010, USA; College of Medicine, University of Nebraska Medical Center (UNMC), Omaha, NE 68198, USA; Department of Pharmacology and Neuroscience, Creighton University, Omaha, NE 68178, USA

**Keywords:** cannabis use, HIV, inhibitory control, somatosensory gating, magnetoencephalography

## Abstract

The main psychoactive component in cannabis—Δ^9^-tetrahydrocannabinol—is known to have anti-inflammatory properties and to alter gamma oscillations, pointing to its potential as a therapeutic agent for people with HIV (PWH). However, it remains unknown how cannabis use among PWH interacts with the neural circuitry underlying inhibitory processing. Herein, using a cross-sectional study design, we collected data from 108 cannabis users and non-users with and without HIV. Participants were interviewed regarding their substance use history and completed a paired-pulse somatosensory stimulation paradigm during magnetoencephalography (MEG). MEG data were imaged using a beamformer and peak voxel time series data were extracted to examine neural oscillations in response to the stimulation and the strength of spontaneous activity in the same tissue during the baseline period. Across all participants, we observed robust gamma oscillations following stimulation in the left primary somatosensory cortices, with responses to the second stimulation being strongly attenuated relative to the first, thus demonstrating somatosensory gating. PWH who used cannabis exhibited stronger oscillatory gamma activity compared with non-users with HIV, while the latter group also exhibited elevated spontaneous gamma activity relative to all other groups. Finally, we found that a longer duration of time since HIV diagnosis was associated with less efficient inhibitory processing among PWH who did not use cannabis, but not among PWH who regularly use cannabis. These findings provide new evidence that cannabis use may mitigate the harmful effects of HIV on oscillatory and spontaneous gamma activity serving inhibitory processing.

## Teaser

Schantell *et al*. report that cannabis use among PWH is associated with normalized spontaneous and oscillatory gamma activity supporting inhibitory processing in response to somatosensory stimulation. Moreover, they show that cannabis may mitigate the impact of HIV on inhibitory processing, suggesting that cannabis may have potential therapeutic properties among PWH.

## Graphical Abstract

**Graphical Abstract:**
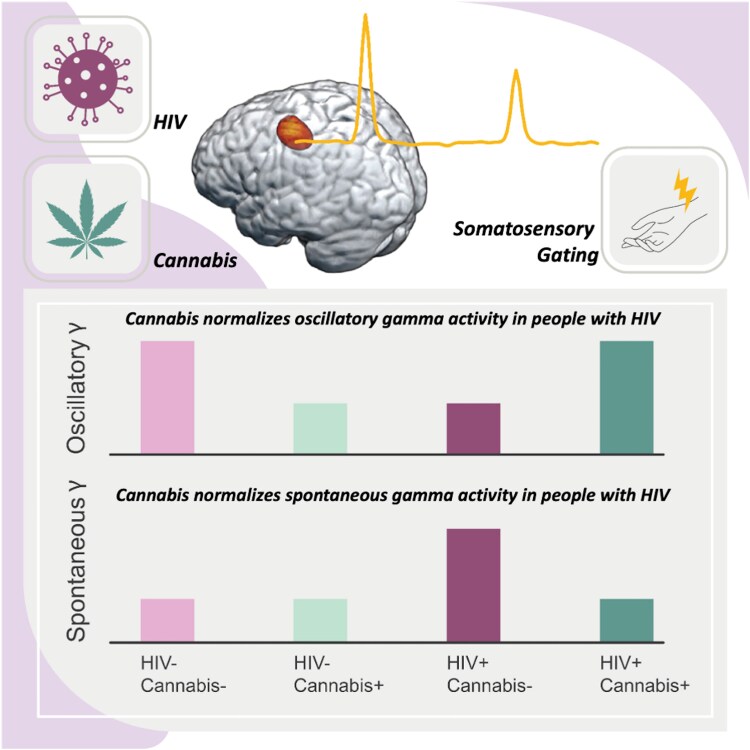

## Introduction

Recent evidence suggests that cannabis has systemic anti-inflammatory properties which may reduce the elevated levels of inflammation that are common among people with HIV (PWH),^1-3^ thus indicating therapeutic potential in at least some clinical populations. Cannabis contains a psychoactive component known as Δ^9^-tetrahydrocannabinol, which is an agonist of the endocannabinoid CB_1_ and CB_2_ receptors.^4^ Interestingly, CB_2_ receptors are located primarily on immune cells, which regulate inflammatory cytokine cascades.^5,6^ In contrast, CB_1_ receptors are found on both glutamatergic and GABAergic neurons, but much more densely on the latter which may indicate that CB_1_ receptors are important for modulating gamma oscillations.^7,8^ More specifically, gamma oscillations are known to be generated by local networks of fast-spiking parvalbumin-expressing GABAergic interneurons, as demonstrated by numerous cellular studies.^9-20^ Translational work in humans has supported such findings, suggesting that local GABA concentrations measured using magnetic resonance spectroscopy are tightly coupled with higher peak gamma frequencies in the same cortical regions.^21-24^

Identifying the impact of regular cannabis use on the neural oscillatory dynamics serving cognition is of critical interest and this is especially true in the context of HIV. Essentially, prior work has illuminated HIV-related alterations in both resting and prestimulus spontaneous neural activity, as well as evoked responses and induced neural oscillations across the cortex,^25-32^ with many studies suggesting that persistent inflammation may play a key role.^33-36^ In particular, PWH exhibit pathologically elevated levels of spontaneous theta, alpha, and gamma activity, along with blunted (i.e. less optimal) theta and alpha oscillations.^25,26,28-32,37,38^ Though studies assessing the combined impact of HIV and regular cannabis use are limited, previous work has demonstrated that regular cannabis use in PWH has a normalising effect on multispectral spontaneous and oscillatory deficits,^37,39^ as well as multiple parameters of network-level connectivity measured with functional MRI.^40^ While these studies clearly suggest beneficial effects, no studies to date have examined how chronic cannabis use may affect the inhibitory processes associated with sensory gating in PWH, which are known to be impaired.^25,28,29,31,41-43^

Sensory gating refers to the inhibitory neurophysiological mechanisms that are thought to help filter redundant sensory information and aid in the preservation of neural resources.^29,44^ In the context of somatosensory gating, two identical stimulations are presented in rapid succession and the attenuation of the neural response to the second stimulus relative to the first stimulus is indicative of effective gating, which is thought to be reflective of inhibitory processing.^45-47^ Smaller gating ratios are indicative of optimally suppressed responses to the second (redundant) stimulation, and extensive work has demonstrated that alterations in both the evoked responses and induced neural oscillations underlying this phenomenon are sensitive and reliable markers of neural decrements across a variety of clinical and healthy populations.^25,28,29,47-56^ In particular, studies have separately identified local aberrations in the oscillatory gamma dynamics serving somatosensory gating in PWH^25,28,29^ and cannabis users,^57^ though no studies to date have examined whether regular cannabis use modulates the aberrant gamma dynamics serving somatosensory gating in PWH. Thus, in the present study, we quantify the impact of chronic cannabis use and HIV on the gamma-specific oscillatory dynamics serving sensory gating using a paired-pulse electrical stimulation paradigm in a large sample of cannabis users and non-users with and without HIV. We hypothesize that PWH who do not use cannabis will exhibit elevated spontaneous gamma activity during the baseline period, as well as altered oscillatory gamma responses following stimulation. Further, we hypothesize that cannabis use will have a normalising effect on these aberrations in PWH. Finally, we predict that regular cannabis use will modulate the relationship between clinical markers of HIV health and neural oscillatory activity supporting somatosensory gating among PWH.

## Methods

### Ethical standards

The authors assert that all procedures contributing to this work comply with the ethical standards of the relevant national and institutional committees on human experimentation and with the Helsinki Declaration of 1975, as revised in 2008.

### Participants

The study included 108 participants drawn from a larger cross-sectional investigation on HIV and aging (MH103220). The sample was comprised of 33 HIV- non-users, 36 HIV- cannabis users, 20 HIV + non-users, and 19 HIV + cannabis users who successfully completed a neuropsychological assessment, structured substance use interview, and a somatosensory gating task during magnetoencephalography (MEG). Cannabis users and non-users with HIV were recruited from the University of Nebraska Medical Center’s HIV Clinic, while the control group (both users and non-users) were recruited from the Omaha metropolitan area. To qualify, PWH were required to be on an effective antiretroviral therapy (ART) regimen, verified by an HIV RNA viral load of <50 copies/mL within 3 months of participation. Controls were confirmed to be seronegative at the time of neuropsychological testing using the OraQuick *ADVANCE*® Rapid HIV-1/2 Antibody Test. Cannabis users were eligible for inclusion if they reported using cannabis at least twice weekly over the previous 6 months and had minimal use of other substances (less than monthly). Participants were excluded if they had a history of neurological or psychiatric disorders, head trauma, current pregnancy, history of substance use disorder (for non-users), or any ferrous metallic implants that might interfere with MEG data acquisition. The study protocol was reviewed and approved by the Institutional Review Board, and all participants provided written informed consent after a thorough explanation of the study procedures.

### Cognitive and substance use assessments

Participants completed a comprehensive cognitive battery designed to evaluate key neurocognitive domains linked to HIV-related impairments.^58^ Cannabis users underwent an in-depth substance use evaluation that included the NIDA Quick Screen (Version 1), NIDA-Modified Alcohol, Smoking, and Substance Involvement Screening Test (ASSIST; Version 2), and Module E of the Structured Clinical Interview for the Diagnostic and Statistical Manual, 5th Edition (SCID-5). Additional self-report measures included the Cannabis Use Disorders Identification Test Revised (CUDIT-R) and a customized questionnaire on cannabis use patterns and history. Samples for urinalysis were collected to confirm the absence of recent substance use other than cannabis. Non-users were interviewed on their past and present substance use using a standardized medical history interview.

### MEG experimental paradigm

Participants were seated comfortably in a non-magnetic chair with their head positioned within the MEG sensor array. Somatosensory stimulation was applied to the right median nerve using an external cutaneous stimulator linked to a Digitimer DS7A constant-current stimulator system (Digitimer Ltd, Garden City, UK). At least 80 paired-pulse trials were administered to each participant, with an inter-stimulus interval of 500 ms and a randomized inter-pair interval ranging from 4500 to 4800 ms. Each pulse generated a 0.2 ms constant-current square wave, calibrated to a level 10% above the threshold required to induce a subtle thumb twitch.

### MEG and MRI data acquisition

A MEGIN VectorView MEG system (Helsinki, Finland) with 306 sensors (204 planar gradiometers, 102 magnetometers) was used to record functional MEG data. Data were acquired using a 1 kHz sampling rate and an acquisition bandwidth of 0.1–330 Hz in a single-layer magnetically shielded room equipped with active shielding. Before MEG acquisition, four head positioning indicator coils were attached to the participant’s head and their locations were recorded alongside fiducial and scalp surface points using a three-dimensional (3D) digitizer (FASTRAK, Polhemus Navigator Sciences, Colchester, Vermont). During the recording session, a unique electric current frequency label (e.g. 322 Hz) was passed through each coil, generating a measurable magnetic field that enabled the precise localisation of the coils relative to the MEG sensor array. Structural T1-weighted images were obtained using a Philips Achieva 3.0T X-Series scanner with a 3D-fast-field echo sequence. The sequence parameters included: TR: 8.09 ms; TE: 3.7 ms; field of view: 24 cm; matrix: 256 × 256; slice thickness: 1 mm with no gap; in-plane resolution: 0.9375 × 0.9375 mm; sense factor: 1.5.^59,60^

### MEG and MRI processing

Preprocessing of MEG and MRI data followed established protocols.^45,61^ Structural MRI data were aligned to the anterior and posterior commissures and standardized to a common reference space. MEG data were subjected to environmental noise reduction, and head motion was corrected for using the signal space separation method with a temporal extension (tSSS; correlation limit: 0.950; correlation window duration: 6 s)^62^ in MEGIN’s MaxFilter software. Subsequent analyses were limited to data from the 204 planar gradiometers. Further processing was conducted in BESA (Research: Version 7.1; MRI: Version 3.0; Statistics: Version 2.1). Cardiac and ocular artefacts were identified and removed from the MEG data using signal space projection.^63^

### MEG time-frequency transformation

Following preprocessing, the MEG data were notch-filtered at 60 Hz and divided into 3700 ms epochs (−800 to 2900 ms), with 0.0 s defined as the onset of the first stimulation. The baseline period extended from −700 to −300 ms prior to stimulation onset to minimize potential anticipatory activity, although no anticipatory effects were observed in the final dataset. Artefact rejection was performed using an individualized fixed threshold method that accounted for participant-specific differences in signal distribution for both amplitude and gradient. This is important, as in MEG, the raw signal amplitude is strongly impacted by the distance between the source (i.e. brain) and the MEG sensor array, as the magnetic field strength falls off sharply as the distance from the current source increases. Epochs exceeding the amplitude or gradient threshold were excluded following visual inspection. Artefact-free epochs were transformed into the time-frequency domain using complex demodulation.^64,65^ This method initially applies a fast Fourier transform to convert the signal into the frequency domain, producing a frequency spectrum that reflects power and cross-spectral properties of the original signal. The frequency spectrum is then (de)modulated in a step-wise manner using complex sinusoids at increasing carrier frequencies (i.e. heterodyning), followed by low-pass filtering to minimize spectral leakage. The resulting spectral power estimates for each sensor were then averaged across trials to produce time-frequency plots of mean spectral density. Sensor-level data were normalized to the mean baseline power (i.e. −700 to −300 ms). Time-frequency windows (5 Hz, 10 ms resolution) for subsequent source imaging were identified using non-parametric permutation tests across all participants and the entire array of gradiometers from 10 to 100 Hz.^30,66^

### MEG source imaging

To conduct source-level analysis, MEG data were co-registered with each participant’s structural T1-weighted MRI. The dynamic imaging of coherent sources beamformer was used to estimate oscillatory activity in the time-frequency window of interest for each stimulation (i.e. Stimulation 1 and Stimulation 2) per participant.^67-69^ Beamformer analysis was conducted using a task and baseline period of equal duration and bandwidth,^70^ generating noise-normalized source power maps. The resulting pseudo-*t* units reflect power differences (i.e. active versus baseline) per voxel (resolution: 4 × 4 × 4 mm). Individual beamformer maps were transformed into standardized space and spatially resampled using the same transform that was applied to the native structural images per participant. Source images from both stimulation conditions were averaged for each participant, and whole-brain grand-averaged maps were generated to identify the peak voxel for virtual sensor analysis.

### Peak voxel time series

Virtual sensor time series were extracted from the peak voxel identified in the grand-averaged source images. This was achieved by applying the sensor-weighting matrix from the forward computation to the preprocessed signal vector, generating two orthogonal time series reflecting activity at the site of interest. These virtual sensor data were decomposed into time-frequency space to derive a single temporal envelope for the dominant orientation within the target frequency band identified through the MEG sensor-level statistical analyses. From these data, absolute and relative (baseline-normalized) power time series for the peak voxel per participant. Absolute power was used to examine differences in spontaneous (baseline) response power, while the relative power was used for neural oscillations after normalisation to the baseline. To mitigate the influence of extreme values, participants with values 3 SDs above or below their respective group means were excluded from further analysis.

### Statistical analyses

Group differences and interactions between cannabis use, HIV status, and stimulation condition were assessed using a 2 × 2 × 2 ANOVA. Stimulation (i.e. Stimulation 1 versus Stimulation 2) was treated as a within-subjects factor, while HIV status and cannabis use were treated as between-subjects factors. In addition, a 1 × 4 ANOVA was used to assess for group differences in spontaneous neural activity during the prestimulus baseline period. Continuous demographic and substance use data were assessed using Kruskal-Wallis ANOVAs, pairwise comparisons were conducted using Wilcoxon tests, and categorical comparisons were conducted using Chi-square tests (χ^2^). Subsequent analyses assessing the relationships between cannabis use and clinical metrics of HIV health by group and their interactions were assessed through ANCOVAs. Demographic, behavioural, time series, and cannabis use frequency analyses were conducted in IBM SPSS v.29.

## Results

### Participant characteristics

The four study groups—control non-users, control cannabis users, HIV + non-users, and HIV + cannabis users—did not differ significantly in terms of demographic characteristics (Table 1). Among participants with HIV, key clinical measures, including years since HIV diagnosis, duration on ART, nadir CD4 counts, and current CD4 counts, were comparable between cannabis users and non-users with HIV (Table 1). All PWH met our inclusion criteria for viral suppression (HIV viral load < 50 copies/mL). Additionally, there were no significant group differences in the number of accepted trials by group (*F* = 0.84, *P* = 0.478), and there was not a significant difference in the motor threshold by group (*F* = 0.87, *P* = 0.459).

**Table 1:** Participant demographics and clinical indices

	HIV- non-users *n* = 33	HIV- users *n* = 36	HIV + non-users *n* = 20	HIV + users *n* = 19	*P*-value
**Age (years)**	36.18 (11.21)	35.29 (10.66)	41.35 (11.85)	42.21 (12.30)	0.075
**Sex (Male/Female)**	16/17	21/15	10/10	14/5	0.316
**Dominant hand (Right/Left)**	29/4	33/3	20/0	18/1	0.496
**CUDIT-R Score**	-	13.08 (4.53)	-	13.32 (4.59)	0.860
**CD4 Nadir (cells/µL)**	-	-	269.84 (172.46)	304.23 (184.58)	0.594
**Current CD4 count (cells/µL)**	-	-	771.15 (264.30)	762.89 (353.41)	0.934
**Years since HIV Diagnosis**	-	-	7.53 (4.07)	8.50 (6.04)	0.594
**Years on ART**	-	-	6.38 (4.05)	6.49 (6.58)	0.934

Means and standard deviations are displayed for all continuous variables, and counts are displayed for categorical variables.

ART, Antiretroviral Therapy; CUDIT-R, Cannabis Use Disorders Identification Test, Revised.

### Neural oscillatory responses

**Figure 1:**
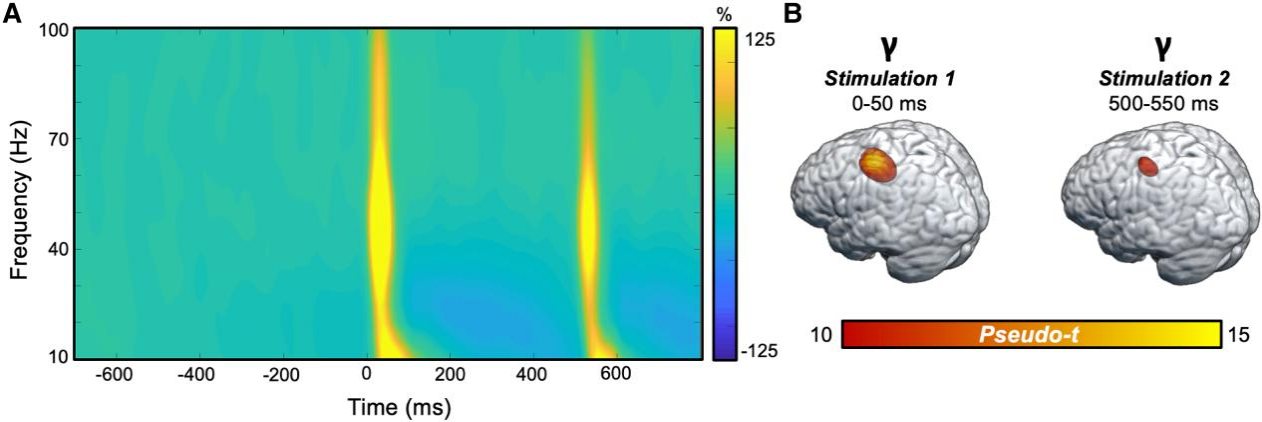
**Time-frequency responses to somatosensory stimulation of the right median nerve.** (**A**) Grand-averaged time-frequency spectrogram of an MEG sensor showing the significant gamma responses (30–90 Hz, stimulation 1: 0–50 ms; Stimulation 2: 500–550 ms) to the stimulations, which occurred at 0 and 500 ms. The spectrogram displays frequency (Hz) on the *y*-axis and time (ms) on the *x*-axis. Signal power is expressed as the per cent difference from the baseline period, with the colour legend shown to the right of the spectrogram. (**B**) Grand-averaged beamformer images (pseudo-*t*) across all participants (*n* = 108) for the gamma time-frequency component for Stimulation 1 (left) and Stimulation 2 (right).

Sensor-level analyses collapsed across all participants revealed two distinct time-frequency windows. Specifically, significant increases in power relative to the baseline period were observed in the gamma (30–90 Hz) band between 0 to 50 ms and between 500 to 550 ms (Fig. 1A). This window was imaged separately for each stimulation (i.e. Stimulation 1 and Stimulation 2) per participant. Whole-brain grand average maps were generated from all participants for Stimulation 1, Stimulation 2, and the average across Stimulations 1 and 2 (Fig. 1B).

### Oscillatory dynamics underlying somatosensory gating

**Figure 2:**
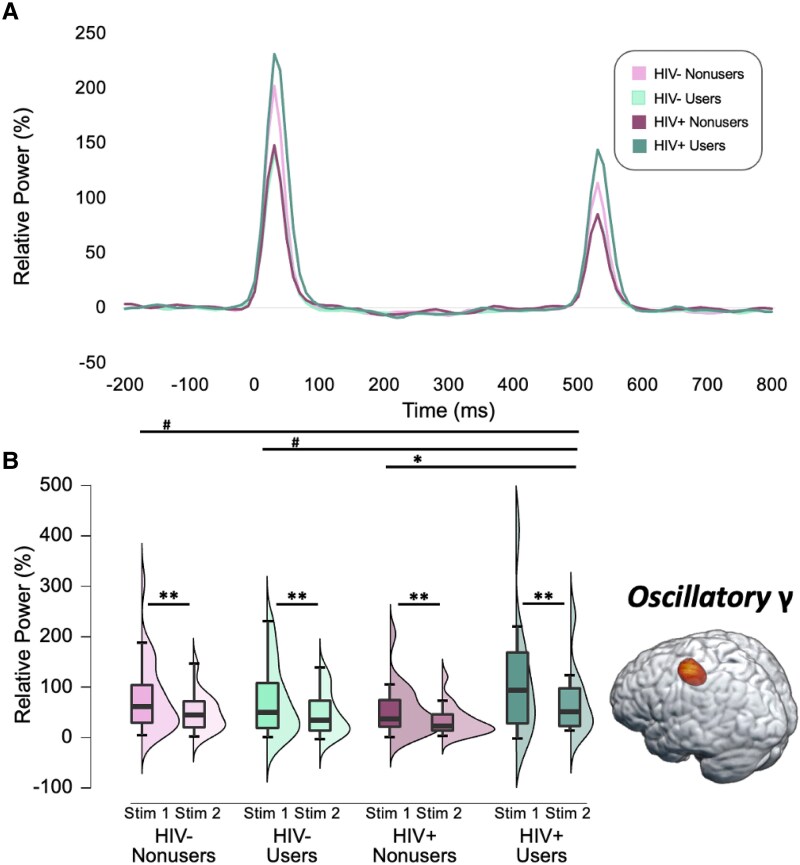
**Regular cannabis use modulates the oscillatory dynamics serving somatosensory gating among PWH.** (**A**) Group averages in the virtual sensor relative gamma power time series (expressed as the per cent change from the baseline period) using a frequency envelope from 30 to 90 Hz. (**B**) In a 2 × 2 × 2 ANOVA across all four groups (*n* = 100; 29 HIV- non-users, 34 HIV- cannabis users, 19 HIV + non-users, 18 HIV + cannabis users), participants exhibited significantly weaker oscillatory gamma responses to the second stimulation relative to the first stimulation in the left primary somatosensory cortex (i.e. the classic somatosensory gating response; *F* = 56.31, *P* < 0.001). Additionally, there was a significant interaction effect (*F* = 4.87, *P* = 0.030) such that PWH who use cannabis had significantly stronger oscillatory gamma responses relative to PWH who did not use cannabis, with marginal differences relative to the other two groups. Raincloud plots display the distribution of relative oscillatory gamma power for each stimulation condition (Stimulation 1 and Stimulation 2) by group, with the corresponding boxplots displaying the median, interquartile range, minimum, and maximum relative gamma power values for each group and stimulation condition. #*P* < 0.080, *P < 0.05, **P < 0.001, γ, Gamma. Stim, Stimulation.

**Figure 3:**
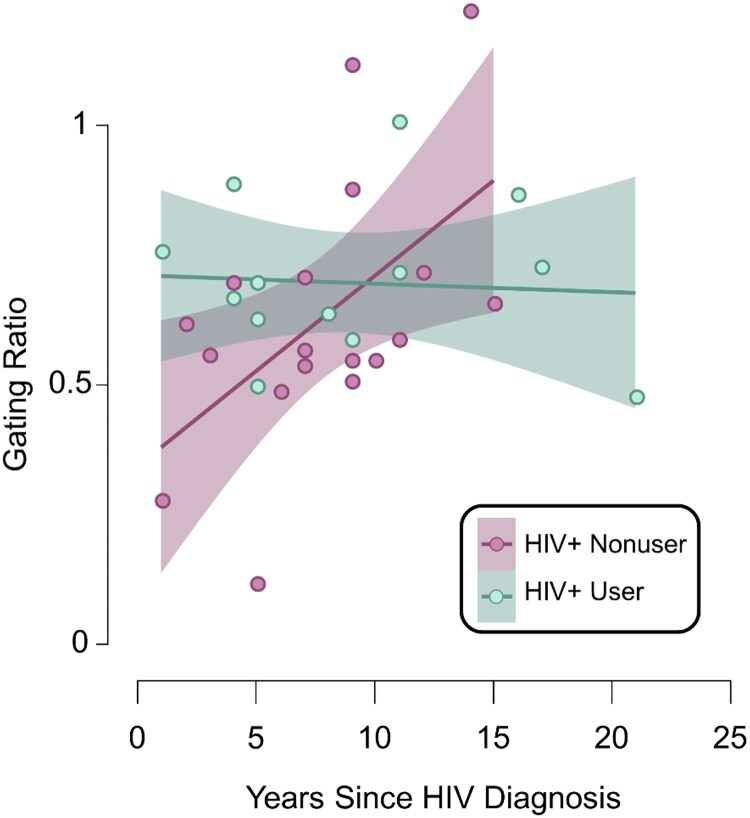
**Cannabis use modulates the relationship between HIV disease duration and somatosensory gating.** An ANCOVA revealed that the number of years since HIV diagnosis was differentially associated with the sensory gating ratio (i.e. oscillatory relative gamma power during the second stimulation divided by that of the first stimulation) in the left primary somatosensory cortex (*F* = 5.29, *P* = 0.029, *n* = 31; 18 HIV + non-users, 13 HIV + cannabis users). Sensory gating became weaker (i.e. closer to 1.0) with increasing disease duration in PWH who did not use cannabis (*n* = 18), while no relationship between disease duration and sensory gating was observed in PWH who regularly use cannabis (*n* = 13). Light magenta points represent the individual gating ratios of non-users with HIV and mint green points represent the gating ratio in cannabis users with HIV. The shaded area surrounding each trendline represents the standard error of the mean.

Next, we extracted voxel times series data from the peak voxel in the left primary somatosensory cortex (Fig. 2A) identified in the whole-brain grand-averaged map across both stimulation conditions and all participants. As described in the methods, we computed the temporal envelope of the signal and then estimated the mean oscillatory power during each window (i.e. 0–50 ms, 500–550 ms), as well as the spontaneous power during the baseline period (i.e. −700 to −300 ms). We then conducted a 2 × 2 × 2 ANOVA with a within-subjects factor of stimulation (i.e. Stimulation 1 versus Stimulation 2), a between-subjects factor of cannabis use status (non-users versus cannabis users), and another between-subjects factor of HIV status (seronegative controls versus PWH) to assess cannabis- and HIV-related differences in the oscillatory dynamics serving somatosensory processing. We found a main effect of stimulation such that oscillatory gamma power was stronger during the first stimulation relative to the second stimulation across all participants (*F* = 56.31, *P* < 0.001). In addition, we observed a cannabis use-by-HIV-status interaction in oscillatory gamma power (*F* = 4.87, *P* = 0.030; Fig. 2B). Follow-up *post-hoc* analyses indicated that PWH who regularly use cannabis had stronger oscillatory gamma responses than PWH who do not use cannabis (*P* = 0.009), as well as marginally stronger oscillatory gamma power relative to seronegative non-users (*P* = 0.078) and cannabis users (*P* = 0.055). We then computed the gating ratio by dividing the average oscillatory power during Stimulation 2 by that of Stimulation 1 per participant and assessed the relationship between sensory gating, cannabis use, and clinical indices of HIV health. Among PWH, we found that regular cannabis use modulated the relationship between the gating ratio and the number of years since being diagnosed with HIV, such that longer durations of HIV disease were associated with weaker gating (i.e. gating ratios closer to 1.0) among PWH who did not use cannabis, while sensory gating was not related to disease duration among PWH who regularly use cannabis (*F* = 5.29, *P* = 0.029; Fig. 3).

### Group differences in spontaneous activity

**Figure 4:**
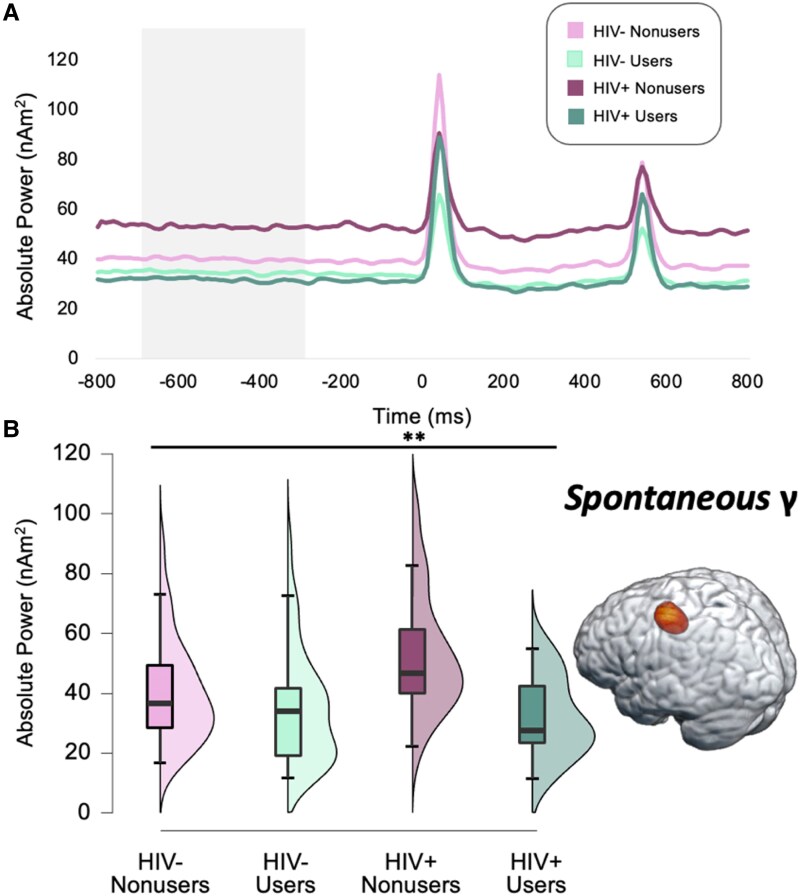
**Spontaneous gamma activity in the left primary somatosensory cortex is affected by HIV.** (**A**) Peak voxel time series of the left primary somatosensory cortex were extracted to estimate spontaneous gamma power (nAm^2^) during the baseline period across all participants (*n* = 106), which differed by group (32 HIV- non-users, 36 HIV- cannabis users, 19 HIV + non-users, 19 HIV + cannabis users). Specifically, a 1 × 4 ANOVA demonstrated that PWH who did not regularly use cannabis had pathologically elevated spontaneous gamma activity relative to the other three groups (*F* = 5.78, *P* = 0.001). (**B**) Raincloud plots show the distribution of spontaneous gamma power during the baseline per group, with the corresponding boxplots displaying the median, interquartile range, minimum, and maximum relative gamma power values for each group. **P  *=* 0.001, γ, Gamma.

To investigate differences in spontaneous gamma power during the baseline (Fig. 4A), we conducted a one-way ANOVA which revealed group differences in the left primary somatosensory cortex (*F* = 5.78, *P* = 0.001; Fig. 4B). *Post-hoc* analyses indicated that PWH who do not use cannabis had sharply elevated spontaneous activity (i.e. more abnormal) relative to cannabis users with and without HIV and controls who do not use cannabis (all *Ps* < 0.013). Finally, we assessed whether spontaneous gamma activity in the somatosensory cortex scaled with cannabis use metrics and/or indices of HIV health, but there were no significant relationships among these measures.

## Discussion

In the present study, we investigated the modulatory impact of regular cannabis use and HIV on the spontaneous and oscillatory gamma dynamics supporting somatosensory gating using a mapping approach with MEG. Our main findings indicated that there were transient broadband gamma responses in the left primary somatosensory cortex in response to paired-pulse electrical stimulation of the right median nerve. Further, we found that regardless of HIV or cannabis use status, participants exhibited significantly weaker oscillatory gamma responses to the second stimulation relative to the first stimulation, thereby exhibiting the classic somatosensory gating response. Importantly, we found that PWH who regularly use cannabis had the strongest oscillatory gamma responses following stimulation. In addition, we directly assessed the modulatory impact of regular cannabis use on the relationship between neural somatosensory gating and clinical indices of HIV health and found that a longer HIV disease duration was associated with progressively weaker sensory gating in PWH who did not regularly use cannabis, while no such relationship was observed in those who use cannabis. Finally, we found that PWH who do not use cannabis had stronger spontaneous gamma activity in the primary somatosensory cortices relative to the other three groups. Such elevated spontaneous activity has been repeatedly linked to neuropathology. Below, we discuss the implications of these findings for understanding the impact of HIV status and cannabis use on inhibitory processing during sensory gating.

Recent work has supported the notion that the potential neuroprotective and therapeutic properties of cannabis may curb the deleterious impacts of HIV on the intricate neural circuitry serving cognition.^37,39,40,71^ To further probe whether cannabis may have a neuroprotective effect in PWH, we quantified spontaneous gamma activity in the left primary somatosensory cortex and found that regular cannabis use was associated with weaker (i.e. more normal) spontaneous gamma activity in PWH, whereas PWH who did not use cannabis had aberrantly elevated spontaneous gamma activity in this region. Previous studies have linked such elevated spontaneous activity to aging and brain pathology in multiple diseases, with extensive work in the area of neuroHIV.^25,26,28-30,48,72,73^ In fact, previous studies have shown that spontaneous activity levels in the cortex can distinguish PWH and controls,^25,26,28-30^ as well as cognitively impaired from unimpaired PWH.^26,30^ Thus, our current finding that cannabis use has a normalising effect on spontaneous gamma activity in PWH reflects a positive change in brain function that may be attributable to the neuroprotective properties of cannabis and is consistent with prior work in PWH, including studies focusing on the visual cortex and prefrontal association regions.^37,39^

Beyond our spontaneous findings, we also found that regular cannabis use was associated with stronger gamma oscillations in PWH. Prior work has found disease-related reductions in oscillatory gamma activity underlying somatosensory gating among PWH.^29^ Thus, our current findings suggest that regular cannabis use among PWH may either preserve or enhance oscillatory gamma activity. This pattern of results may reflect long-term changes in the distribution and/or action of CB_1_ receptors^7,8^ in PWH who use cannabis, but additional work is needed to fully understand the underlying mechanisms and the importance of these changes for cognitive processing. Aberrant gamma oscillations have been widely reported in PWH,^28,31,51,74-76^ with deficits identified across multiple brain areas serving a collection of different functions. Therefore, the capacity to normalize gamma oscillations in PWH could have a major impact in this population and should be a focus of future studies.

Substantial evidence suggests that gamma oscillations originate from the interaction of GABAergic fast-spiking parvalbumin inhibitory interneurons and pyramidal cells.^9,11-19,77^ Chronic cannabis use may affect such gamma oscillations by modulating CB_1_ receptors on GABAergic interneurons,^7,78-82^ which is supported by previous work linking chronic cannabis use to the downregulation and desensitisation of CB_1_ receptors across the cortex.^83,84^ Studies using MEG and EEG have corroborated these findings, demonstrating that regular cannabis use is associated with altered gamma oscillations both at rest and during task performance.^39,57,72,85-87^ Presumably, the cannabis-related changes observed in PWH reflect a similar mechanism, although greater involvement of CB_2_ receptors on immune cells is also likely.

Though the work presented herein possesses many strengths, there are some limitations of this study that should be addressed in future work. In particular, we did not measure markers of inflammation in the present study, and thus, we cannot conclude whether our findings provide evidence in support of or against the theory that cannabis use has anti-inflammatory effects on HIV-related systemic inflammation.^1-3^ Future studies should assess the impact of regular cannabis use on systemic inflammatory processes among PWH, and further, how cannabis-induced reductions in inflammation scale with the neural oscillatory activity serving cognitive functioning. Such an approach is especially promising, as we recently demonstrated significant coupling between specific inflammatory markers and neural oscillations in a normative sample of adults^88^ and showed that inflammation is also associated with cognitive function in PWH.^35^

Additionally, we did not record the time of the MEG recordings, nor did we record when cannabis users last used cannabis, which may have impacted the results of this study.^89^ Therefore, future work should control for circadian dynamics in oscillatory activity and evaluate the effects of the duration since participants last used cannabis on the neural dynamics supporting inhibitory processing. Of note, participants in the cannabis use groups in the current study were instructed not to consume cannabis on the day of their visit, but cannabis use on the day prior to their visit could still have residual effects on neural activity during the recording, thus underscoring the importance of future work in this area. Although beyond the scope of the present study, future work should integrate both structural and functional measures to provide a more comprehensive understanding of how cannabis and HIV interact to influence the relationship between cortical structure and inhibitory processing in the somatosensory cortices, as prior normative studies have shown strong structure-function coupling.^55^

Further, while our sample sizes of cannabis users and non-users with HIV were sufficient for MEG analyses, we were limited in our ability to assess various aspects of cannabis use such as the frequency and duration of use, as well as the methods of use, and their relationships with gamma activity in the primary somatosensory cortex. Future studies should enroll larger numbers of cannabis users who have a broader distribution of use to enable these cannabis use parameters to be linked with neural responses. Along these lines, PWH who use cannabis appeared to exhibit greater variability in their relative gamma power following the first median nerve stimulation, which may reflect greater heterogeneity in these responses. Future studies focused on this seemingly greater variability and the underlying causes are also warranted. Finally, there may have been group differences in habituation to the paired electrical stimuli that should be investigated in future work. Despite these limitations, our study revealed the modulatory impact of regular cannabis use on the spontaneous and oscillatory gamma dynamics serving inhibitory processing in the somatosensory cortices among those with and without HIV.

Taken together, these findings suggest that regular cannabis use may have a neuroprotective effect on inhibitory processing in PWH by normalising spontaneous gamma activity and enhancing gamma oscillatory responses during sensory gating. This pattern indicates that cannabis use could potentially mitigate some of the neural disruptions associated with HIV, highlighting a promising target for future interventions aimed at preserving cognitive function in this population. Importantly, the capacity of cannabis to influence gamma dynamics underscores the broader role of the endocannabinoid system in shaping neural function in the context of HIV-related neuropathology. Future work should aim to disentangle the complex mechanisms underlying these effects and evaluate whether cannabis-related improvements in neural function translate into measurable gains in cognitive and sensory processing outcomes.

## Data Availability

De-identified data supporting the findings of this study are available upon request from the corresponding author. Data have also been deposited in the Collaborative Informatics and Neuroimaging Suite database for public access.
